# Disseminated cutaneous histoplasmosis with laryngeal involvement in a setting of immune reconstitution inflammatory syndrome

**DOI:** 10.4102/sajhivmed.v18i1.693

**Published:** 2017-04-28

**Authors:** Mohamed F. Sacoor

**Affiliations:** 1 Department of Dermatology, Nelson R Mandela School of Medicine, University of KwaZulu-Natal, South Africa

## Abstract

**Introduction:**

Histoplasmosis is a systemic mycosis caused by the dimorphic fungus *Histoplasma capsulatum*. We report a case of disseminated cutaneous histoplasmosis with mucocutaneous involvement in an AIDS patient paradigmatic of the multifaceted nature of the disease, which is an expression of the immune reconstitution inflammatory syndrome (IRIS).

**Patient presentation:**

A 39-year-old man presented with a three month history of asymptomatic papules and nodules with necrotic centres involving the centrofacial region. The patient was diagnosed as being HIV-positive a month earlier and was commenced on antiretroviral treatment. Two weeks after the development of skin lesions, the patient complained of a sore throat and hoarseness of his voice. A fibre-optic laryngoscopy and biopsies of the skin, larynx and liver were performed.

**Management and outcome:**

The CD4 counts increased from 2 cells/µL to 124 cells/µL, whereas the viral load decreased from one million to less than 20 copies/mL. A fibre-optic laryngoscopy revealed a supraglottitis with ulceration on the epiglottis. Histology of the liver, larynx and sections of the skin demonstrated pandermal necrotising granulomatous inflammation. Grocott-Gomori methenamine silver and Periodic acid–Schiff (PAS) stains revealed a relative paucity of intracellular, narrow-neck budding fungal organisms. Culture findings confirmed the diagnosis of histoplasmosis. The patient was treated with intravenous amphotericin B for two weeks followed by oral itraconazole 100 mg twice a day, with an excellent response to treatment.

**Conclusion:**

We present this case to remind clinicians that disseminated histoplasmosis in AIDS patients may occur as an expression of IRIS. A sudden onset of hoarseness with cutaneous lesions in a patient with disseminated disease should alert one to possible laryngeal histoplasmosis. Prompt recognition and treatment will avert the potential fatal complications of this disease.

## Introduction

Histoplasmosis is a systemic mycosis caused by the dimorphic fungus *Histoplasma capsulatum*. We report a case of disseminated histoplasmosis (DH) with mucocutaneous involvement in an AIDS patient paradigmatic of the multifaceted nature of the disease, which is an expression of the immune reconstitution inflammatory syndrome (IRIS).

## Ethical considerations

The sub-committee of the Biomedical Research Ethics Committee at the University of Kwazulu-Natal has given their full ethical approval for this study with BREC reference number: BE068/17.

## Case report

A 39-year-old man presented with a 3-month history of skin lesions involving the face, neck and extremities. The patient was diagnosed as being HIV-positive a month earlier and was commenced on antiretroviral treatment abacavir 300 mg bd, lamivudine 300 mg bd and efavirenz 600 mg once daily, because of impaired renal function or pre-renal failure. The patient was hydrated with an improvement in the renal function. Two weeks after the development of the skin lesions, the patient complained of a sore throat and hoarseness of his voice.

**FIGURE 1:**
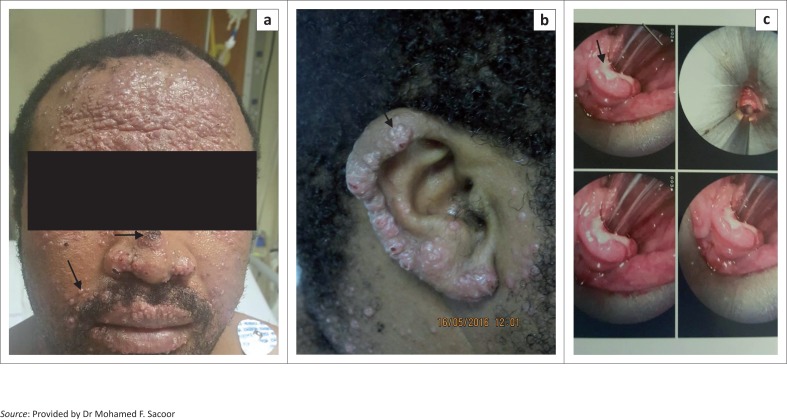
(a) Multiple pearly papules and nodules with necrotic centres on the face and neck, (b) well-circumscribed verrucous papules and plaques on the ear and (c) a supraglottitis with ulceration on the lingual surface of the epiglottis.

On clinical examination, the patient had multiple translucent, clustered, umbilicated papules and nodules in the centrofacial region and neck with necrotic centres. In addition, there were verrucous plaques and nodules on the ears and upper limbs (Figure 1a and b). Chest X-ray was normal with no lung, hilar or mediastinal lymph nodes. A fibre-optic laryngoscopy revealed a supraglottitis with ulceration on the lingual surface of the epiglottis (Figure 1c).

**FIGURE 2:**
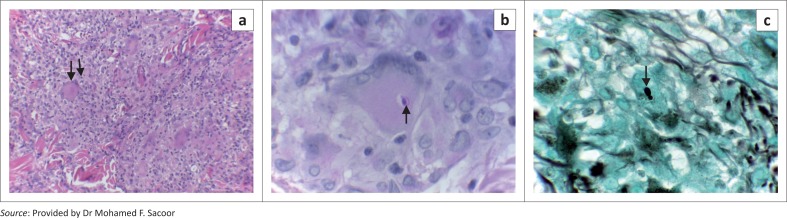
(a) Haematoxylin–eosin 40x – shows features of a pandermal granulomatous inflammatory reaction comprising aggregates of epithelioid histiocytes and Langerhans giant cells; (b) PAS stain 40x reveals the presence of intracellular uninucleate oval yeast-like, narrow-neck budding fungal organisms; and (c) Grocott-Gomori methenamine silver stain 40x – shows a narrow-neck budding fungal organism.

Routine blood investigations demonstrated elevation of the liver enzymes with an infiltrative pattern (AST-63/ALT 70/ALP -219/Gamma GT-291). Abdominal ultrasound revealed an enlarged liver with abnormal parenchyma. The kidneys and the rest of the abdomen were normal. The initial CD4 count performed prior to the commencement of antiretrovirals was 2 cells/µL with a viral load greater than one million copies/mL. A repeat test (eight weeks later) showed a CD4 count of 124 cells/µL with a viral load of less than 20 copies/mL. Urinalysis and urine histoplasma antigen test were negative. Histology of the liver, larynx and sections of the skin demonstrated pandermal necrotising granulomatous inflammation. Grocott-Gomori methenamine silver and PAS stains revealed a relative paucity of intracellular oval, narrow-neck budding fungal organisms morphologically in keeping with *Histoplasma capsulatum var. capsulatum* (Figure 2a and b). Fungal culture of liver, epiglottis ulcer and skin confirmed the diagnosis of histoplasmosis.

On the basis of the clinical presentation, initial worsening of the disease and rapid elevation of the CD4+ T-cell count with lowering of the HIV viral load, the patient most likely has histoplasmosis associated IRIS. The patient was commenced on intravenous amphotericin B (0.8 mg/kg/day) for two weeks and continued with his antiretroviral treatment. He had an excellent response to this treatment and was discharged on itraconazole 100 mg twice a day.

## Discussion

Disseminated histoplasmosis is most frequently described among patients with CD4+ T-cell counts below 50 cells/mm^3^.^1^ It occurs in 5% – 75% of AIDS patients, with mucocutaneous manifestations seen among 11% – 25% of cases.^2^ Cutaneous lesions can take a number of forms from inflammatory folliculitis, molluscum-like papules, verrucous plaques, erythema multiforme-like lesions, vasculitic lesions, exfoliative dermatitis, ulcers and nodular lesions.^2,3^

Immune reconstitution inflammatory syndrome is an inflammatory disease and is the consequence of an exaggerated dysregulated immune antigen interaction following highly active antiretroviral therapy (HAART) induced immune restoration.^4^ The disseminated clinical presentation may probably be due to a low CD4 count as well as a high antigen burden prior to HAART initiation.^4^ Nacher et al.^5^ reported that patients taking HAART were more likely to develop DH than untreated patients. The exacerbation of skin and laryngeal symptoms in our patient after starting HAART may be due to an exaggerated cell-mediated inflammatory response. IRIS is a diagnosis of exclusion. Although it is the most likely diagnosis in our patient, the natural progression of a pre-existing opportunistic infection cannot be excluded.^5^

Laryngeal histoplasmosis is a rare phenomenon.^6^ Since 1952, when laryngeal histoplasmosis was initially described in the literature, less than 100 cases have been reported to date.^7^ Common initial manifestations are pain when swallowing, hoarseness, gingival ulceration and dysphagia.^7,8^ Firm, painful ulcers, with elevated borders, involving the oral mucosa and larynx are characteristic.^7,8^

Amphotericin B and itraconazole are the antifungal agents that were noted to be effective in the treatment of histoplasmosis.^9^ Treatment should be continued until clinical and laboratory findings are normal.^9,10^ However, 9% of patients will experience a relapse.^10^

According to the evidence-based guidelines for the management of DH presented by the Infectious Diseases Society of America (IDSA), it is recommended that patients with moderate to severe disease be treated with liposomal amphotericin B (3.0 mg/kg daily for 12 weeks), followed by oral itraconazole (200 mg three times daily for three days and then 200 mg twice daily for a total of at least 12 months).^11^ The deoxycholate formulation of amphotericin B (0.7 mg/kg – 1.0 mg/kg daily) is an alternative to a lipid formulation in patients who are at low risk for nephrotoxicity. For mild to moderate disease, itraconazole (200 mg three times daily for three days and then 200 mg twice daily for at least 12 months) is recommended.^11^

Lifelong suppressive therapy with itraconazole (200 mg daily) may be required in immunosuppressed patients if immunosuppression cannot be reversed and in patients who relapse despite receipt of appropriate therapy.^11^

## Conclusion

We are presenting this case to remind clinicians that DH in AIDS patients may occur as an expression of IRIS. A sudden onset of hoarseness with cutaneous lesions in a patient with disseminated disease should alert one to possible laryngeal histoplasmosis. Prompt recognition and treatment will avert the potential fatal complications of this disease.
